# Prevalence of *Borrelia burgdorferi*, *Anaplasma* spp., *Ehrlichia* spp. and *Dirofilaria immitis* in Canadian dogs, 2008 to 2015: a repeat cross-sectional study

**DOI:** 10.1186/s13071-019-3299-9

**Published:** 2019-01-28

**Authors:** Michelle Evason, Jason W. Stull, David L. Pearl, Andrew S. Peregrine, Claire Jardine, Jesse S. Buch, Zachary Lailer, Tom O’Connor, Ramaswamy Chandrashekar, J. Scott Weese

**Affiliations:** 1 University of Prince Edward Island, Charlottetown, Prince Edward Island C1A 4P3 Canada; 2 University of Guelph, Guelph, Ontario N1G 2W1 Canada; 3 The Ohio State University, Columbus, Ohio 43210 USA; 4 IDEXX Laboratories, Inc., 1 IDEXX Drive, Westbrook, Maine 04092 USA

**Keywords:** Lyme, Vector-borne, Tick-borne, Heartworm, Co-infection

## Abstract

**Background:**

Vector-borne pathogens are emerging concerns in multiple regions of Canada. Determining regional prevalence of canine vector-borne pathogens and documenting change will improve clinician awareness, enable targeted prevention, enhance diagnosis and ideally reduce the risk of disease. Study objectives were to: (i) estimate the prevalence of positive canine vector-borne test results from samples submitted in Canada; (ii) assess change in prevalence over time, from baseline (2008) to 2015; and (iii) estimate the prevalence of pathogen co-infections.

**Methods:**

This repeat cross-sectional study evaluated 753,468 test results for *D. immitis* antigen and *B. burgdorferi*, *Ehrlichia canis/ewingii/muris* serology, and 753,208 test results for *Anaplasma phagocytophilum/platys* serology using the SNAP® 4Dx®Test and SNAP 4Dx® Plus Test.

**Results:**

Based on all submitted samples from Canada (2008–2015), the period seroprevalence of *B. burgdorferi*, *Ehrlichia* spp., *Anaplasma* spp. and *D. immitis* antigen were 2.0%, 0.5%, 0.4% and 0.2%, respectively. Over the 7 years (2008 compared to 2015) we observed a significant increase in seroprevalence for *B. burgdorferi* (144.4%) and *Ehrlichia* spp. (150%). Co-infections (positive for two or more pathogens on a single 4 pathogen test kit) were estimated at 5.4% (1162/21,612) of total positive tests.

**Conclusions:**

The temporal rise and geographical differences in prevalence detected for these pathogens (notably *B. burgdorferi*) are consistent with anecdotal information on canine illness related to tick-borne pathogen exposure in multiple regions of Canada, particularly canine Lyme disease.

**Electronic supplementary material:**

The online version of this article (10.1186/s13071-019-3299-9) contains supplementary material, which is available to authorized users.

## Background

Geographical ranges of ticks and their associated vector-borne pathogens, notably *Ixodes scapularis* and the spirochete *Borrelia burgdorferi*, are rapidly expanding in central, eastern and Atlantic Canada [1–6]. There is increasing concern over the emergence of tick-borne pathogens, such as *B. burgdorferi*, *Anaplasma* spp. and *Ehrlichia* spp. and the diseases they can cause in dogs [7–9]. Clinical signs related to these tick-borne pathogens are frequently vague and may include fever and shifting limb lameness [7–11]. However, severe clinicopathologic changes and disease can occur, such as Lyme nephritis [8, 10], thrombocytopenia or death due to *Ehrlichia* or *Anaplasma* spp. [9, 11].

Testing for *B. burgdorferi*, *Anaplasma* and *Ehrlichia* spp. serostatus is commonly performed in dogs in conjunction with annual heartworm (*D. immitis*) screening. This widespread testing provides abundant prevalence data and an avenue for surveillance through analyses of large datasets of reported test results for vector-borne pathogen exposure and infection in dogs. This technique has been used to monitor for pathogen prevalence in dogs in the USA and Canada over the past decade [5, 12, 13]. Reported prevalence of canine vector-borne pathogens in Canada has varied by study, with respect to variation in geography (local and regional), year performed and test methodology [5, 14–18]. One publication using data contained within our study period (2013–2014), estimated national canine seroprevalence for *B. burgdorferi* as 2.5% based on 115,636 tests [5].

Mosquitoes transmit *D. immitis* larvae, which can develop into adult worms and lodge in the canine pulmonary system leading to severe pathology and death without therapy. In Canada, heartworm prevalence is currently considered low (< 0.5 %) [5]; however, regional prevalence estimates have varied [5, 15].

Our aims were to investigate the overall prevalence of positive test results for *B. burgdorferi*, *E. canis/ewingii/muris*, *A. phagocytophilum/platys* and *D. immitis* in dogs across Canada tested using a widely used commercial assay, assess broad-scale temporal (annual) and spatial (province) change, and provide a current estimate of prevalence and co-infection prevalence in Canada.

## Results

A total of 753,468 test results were available for *B. burgdorferi*, *Ehrlichia* spp. and *D. immitis*, with results for *Anaplasma* spp. available for 753,208. Tests were predominantly from Ontario (ON) 75% (*n* = 564,552), Quebec (QC) 13% (*n* = 95,898) and Manitoba (MB) 9.6% (*n* = 72,254). The remaining provinces and territories each consisted of < 1% (*n* = 20,764) of test results (Table 1). No results were obtained from the Northwest Territories or Nunavut. Prince Edward Island (PE) and the Yukon were excluded from provincial comparisons due to small sample sizes (*n* = 3 and *n* = 12, respectively). There was a 2142% increase in annual test submissions over the study period, from 8082 (2008) to 181,205 (2015).

**Table 1:** Canine vector-borne pathogen seroprevalence in Canada (2008–2015) for *Borrelia burgdorferi*, *Ehrlichia* and *Anaplasma* spp., and the antigen of *Dirofilaria immitis* (*n* = 753,468), and univariable associations between province and serostatus

Province		*B. burgdorferi*	*Ehrlichia* spp.	*Anaplasma* spp.	*D. immitis*
Ontario	No. positive/No. tested (%)	10,186/564,552 (1.8)	2009/564,552 (0.4)	1719/564,476 (0.3)	703/564,552 (0.1)
	Odds ratio	Referent	Referent	Referent	Referent
Alberta	No. positive/No. tested (%)	54/6697 (0.8)	329/6697 (4.9)	98/6664 (1.5)	12/6,697 (0.2)
	Odds ratio; *P*-value	0.4; *P* < 0.001	14.5; *P* < 0.001	4.9; *P* < 0.001	1.4; *P* = 0.2
British Columbia	No. positive/No. tested (%)	23/4815 (0.5)	316/4815 (6.6)	133/4802 (2.8)	7/4815 (0.2)
	Odds ratio; *P*-value	0.3; *P* < 0.001	19.7; *P* < 0.001	9.3; *P* < 0.001	1.2; *P* = 0.6
Manitoba	No. positive/No. tested (%)	1718/72,254 (2.4)	211/72,254 (0.3)	695/72,158 (1.0)	250/72,254 (0.4)
	Odds ratio; *P*-value	1.3; *P* < 0.001	0.8; *P* = 0.006	3.2; *P* < 0.001	2.8; *P* < 0.001
New Brunswick	No. positive/No. tested (%)	158/2129 (7.4)	17/2129 (0.8)	18/2129 (0.9)	3/2129 (0.1)
	Odds ratio; *P*-value	4.4; *P* < 0.001	2.3; *P* = 0.001	2.8; *P* < 0.001	1.1; *P* = 0.8
Newfoundland	No. positive/No. tested (%)	6/236 (2.5)	3/236 (1.3)	1/236 (0.4)	1/236 (0.4)
	Odds ratio; *P*-value	1.4; *P* = 0.4	3.6; *P* = 0.03	1.4; *P* = 0.7	3.4; *P* = 0.2
Nova Scotia	No. positive/No. tested (%)	592/6464 (9.2)	37/6464 (0.6)	85/6429 (1.3)	12/6464 (0.2)
	Odds ratio; *P*-value	5.5; *P* < 0.001	1.6; *P* = 0.004	4.4; *P *< 0.001	1.5; *P* = 0.2
Prince Edward Island	No. positive/No. tested (%)	1/3 (33.3)	0/3 (0)	0/3 (0)	0/3 (0)
	Odds ratio; *P*-value	np	np	np	np
Quebec	No. positive/No. tested (%)	2229/95,898 (2.3)	582/95,898 (0.6)	363/95,891 (0.4)	274/95,898 (0.3)
	Odds ratio; *P*-value	1.3; *P* < 0.001	1.7; *P* < 0.001	1.2; *P* < 0.001	2.3; *P* < 0.001
Saskatchewan	No. positive/No. tested (%)	3/408 (0.7)	5/408 (1.2)	0/408 (0)	1/408 (0.3)
	Odds ratio; *P*-value	0.4; *P* = 0.1	3.4; *P* = 0.006	np	2.0; *P* = 0.5
Yukon	No. positive/No. tested (%)	0/12 (0)	1/12 (8.3)	0/12 (0)	0/12 (0)
	Odds ratio; *P*-value	np	np	np	np
All of Canada	No. positive/No. tested (%)	14,970/753,468 (2.0)	3510/753,468 (0.5)	3112/753,208 (0.4)	1263/753,468 (0.2)

*Notes*: Reported values are test results not individual dogs. Odds ratio and associated *P*-value reported for logistic regression analysis between positive test result (outcome) and province (Prince Edward Island and Yukon excluded due to low cell counts); all four models had overall *P* < 0.0001

*Abbreviation*: *np* not performed due to low cell counts

### Nationwide prevalence

Over the 8-year period, 2.0% of all samples were positive for *B. burgdorferi,* 0.5% for *Ehrlichia* spp., 0.4% for *Anaplasma* spp. and 0.2% for *D. immitis* (Table 1). Overall, there were significant geographical differences in pathogen period prevalence (Table 1) and increasing trends in percent positive results in dogs from Ontario, Manitoba and Quebec over the study timeframe for all tick-borne pathogens (Additional files 1, 2, 3: Figures S1-S3), with marked positive change for *B. burgdorferi*. Annual seroprevalence for *B. burgdorferi* significantly increased (144% increase) over the study timeframe, from 0.9% to 2.2% (Cuzick test of trend: *Z* = 17.10, *P* < 0.001; Table 2). Similarly, there was a significant increase (150% increase) in *Ehrlichia* spp. annual seropositivity (Table 2) from 0.2% to 0.5% (Cuzick test of trend: *Z* = 10.98, *P* < 0.001). Nationally, significant linear trends were not detected in the annual prevalence of *D. immitis* or *Anaplasma* spp. (Table 2).

**Table 2:** Provincial temporal changes in canine vector-borne pathogen seroprevalence in Canada (2008, 2015) for *Borrelia burgdorferi*, *Ehrlichia* and *Anaplasma* spp., and the antigen of *Dirofilaria immitis*

Province	Year	*B. burgdorferi*			*Ehrlichia* spp.			*Anaplasma* spp.			*D. immitis*		
		Positive (%)^a^	*P* ^b^	Percent change^c^	Positive (%)^a^	*P* ^b^	Percent change^c^	Positive (%)^a^	*P* ^b^	Percent change^c^	Positive (%)^a^	*P* ^b^	Percent change^c^
Alberta	2008	1/253 (0.4)	0.08	25.0	4/253 (1.6)	<0.001	325.0	2/221 (0.9)	0.02	133.3	0/253 (0)	0.1	na
	2015	7/1335 (0.5)			91/1335 (6.8)			28/1335 (2.1)			1/1335 (0.07)		
British Columbia	2008	0/94 (0)	0.8	na	2/94 (2.1)	0.5	176.2	0/81 (0)	0.03	na	0/94 (0)	0.09	na
	2015	4/1201 (0.3)			69/1201 (5.8)			26/1201 (2.2)			4/1201 (0.3)		
Manitoba	2008	23/1713 (1.3)	<0.001	146.2	0/1713 (0)	<0.001	na	5/1619 (0.3)	0.001	233.3	1/1713 (0.06)	<0.001	733.3
	2015	391/12,253 (3.2)			45/12,253 (0.4)			118/12,253 (1.0)			61/12,253 (0.5)		
New Brunswick	2008	1/15 (6.7)	0.08	31.3	0/15 (0)	0.5	na	0/15 (0)	0.2	na	0/15 (0)	0.07	na
	2015	53/604 (8.8)			3/604 (0.5)			8/604 (1.3)			3/604 (0.5)		
Newfoundland	2008	np	0.5	na	np	0.2	na	np	0.2	na	np	0.2	na
	2015	3/55 (5.5)			1/55 (1.8)			1/55 (1.8)			1/55 (1.8)		
Nova Scotia	2008	7/290 (2.4)	<0.001	441.7	1/290 (0.3)	0.3	166.7	0/264 (0)	<0.001	na	0/290 (0)	0.5	na
	2015	194/1490 (13.0)			13/1490 (0.8)			37/1490 (2.5)			0/1490 (0)		
Ontario	2008	31/5018 (0.6)	<0.001	216.7	4/5018 (0.08)	<0.001	400.0	4/4949 (0.08)	0.8	275.0	16/5018 (0.3)	0.1	-33.3
	2015	2799/146,056 (1.9)			627/146,056 (0.4)			424/146,055 (0.3)			213/146,056 (0.2)		
Prince Edward Island	2008	np	0.2	na	np	na	na	np	na	na	np	na	na
	2015	0/1 (0)			0/1 (0)			0/1 (0)			0/1 (0)		
Quebec	2008	6/694 (0.9)	<0.001	233.3	1/694 (0.1)	<0.001	600.0	1/692 (0.14)	0.001	185.7	3/694 (0.4)	<0.001	-25.0
	2015	539/18,068 (3.0)			128/18,068 (0.7)			68/18,068 (0.4)			54/18,068 (0.3)		
Saskatchewan	2008	0/5 (0)	0.8	na	0/5 (0)	0.6	na	0/5 (0)	na	na	0/5 (0)	0.8	na
	2015	1/140 (0.7)			0/140 (0)			0/140 (0)			0/140 (0)		
Yukon	2008	np	na	na	np	0.9	na	np	na	na	np	na	na
	2015	0/2 (0)			0/2 (0)			0/2 (0)			0/2 (0)		
Canada	2008	69/8082 (0.9)	<0.001	144.4	12/8082 (0.2)	<0.001	150.0	12/7846 (0.2)	0.8	100.0	20/8082 (0.3)	0.3	-33.3
	2015	3991/181,205 (2.2)			977/181,205 (0.5)			710/181,204 (0.4)			337/181,205 (0.2)		

^a^Percent Positive = (number positive/number tested) × 100%

^b^*P*-value for non-parametric Cuzick test of annual trend 2008 through 2015; 2009–2014 data not shown

^c^Percent Change = (percent positive 2015 – percent positive 2008)/percent positive 2008 ×100%

*Abbreviations*: na, unable to calculate due to low or zero counts; np, no testing reported

**Fig. 1:**
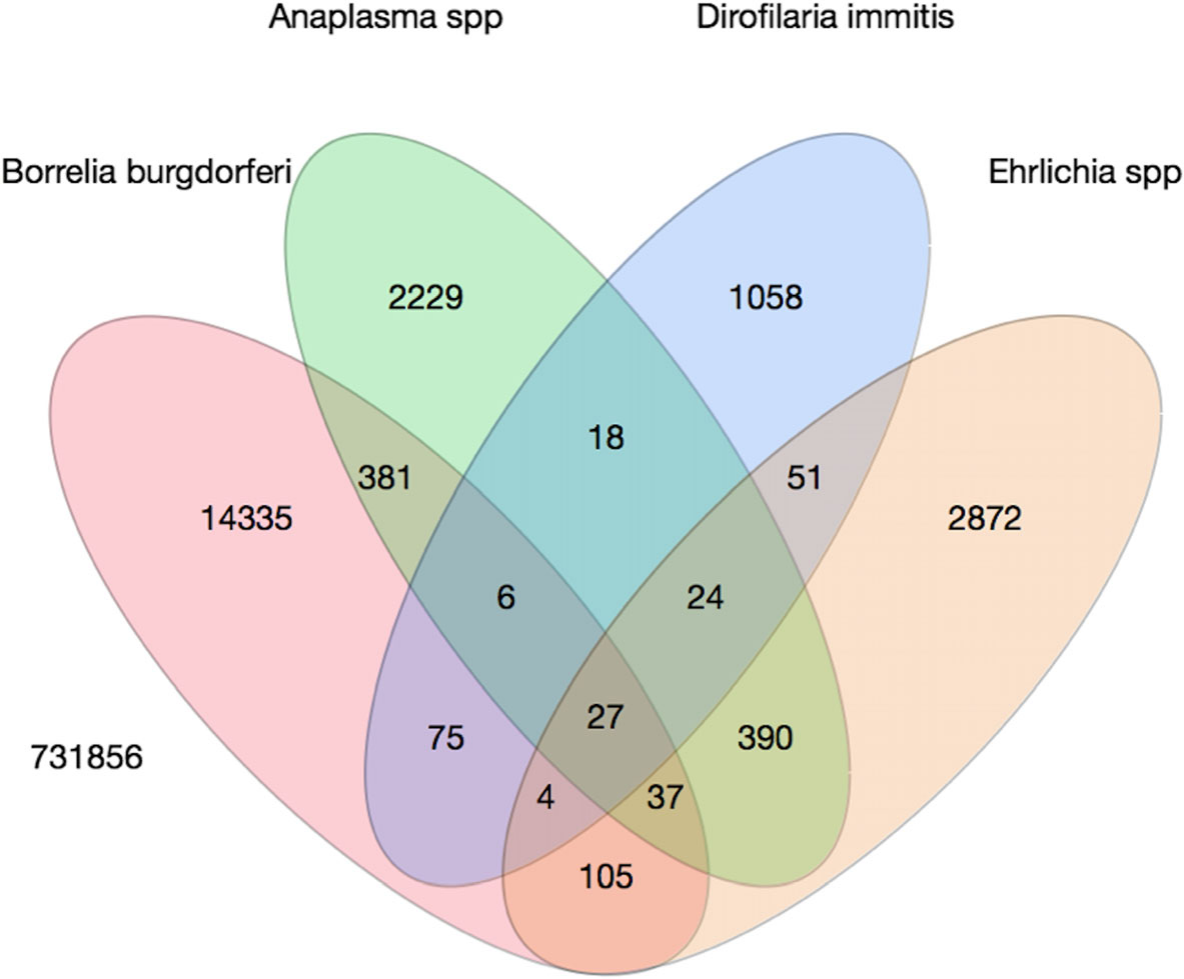
Venn diagram of canine co-infection in Canada (2008–2015), for *Borrelia burgdorferi*, *Ehrlichia* and *Anaplasma* spp., and the antigen of *Dirofilaria immitis* (*n* = 753,468)

Co-infections, defined as a positive result to two or more pathogens from the same sample, were identified in 5.4% (1162) of all positive tests (21,612) (Fig. 1). Co-infections were observed between all pathogens, and most common between: *Anaplasma* and *Ehrlichia* spp. (2.2%; *n* = 478/21,595), *B. burgdorferi* and *Anaplasma* spp. (2.1%; *n* = 451/21,595), and *B. burgdorferi* and *Ehrlichia* spp. (0.8%; 173/21,612).

### Provincial prevalence

**Fig. 2:**
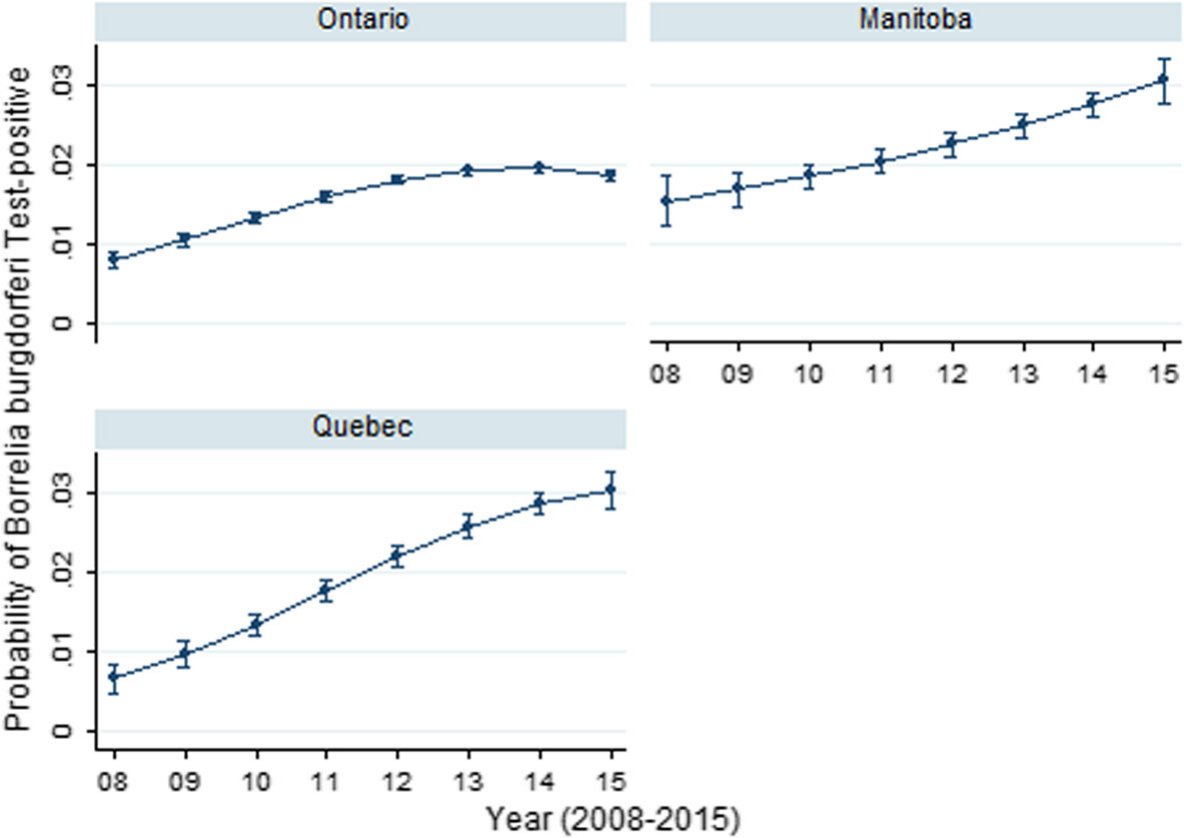
Predictive probability (with 95% CIs) of *Borrelia burgdorferi* positive result on SNAP 4Dx tests performed on canine blood samples from dogs in Ontario, Manitoba and Quebec, Canada (2008–2015)

The seroprevalence of *B. burgdorferi* significantly varied across the provinces (Table 1). The odds of *B. burgdorferi* seropositivity were significantly greater in MB (2.4%), QC (2.3%), Nova Scotia (NS) (9.5%) and New Brunswick (NB) (7.4%) than ON (1.8%; referent). Seroprevalence was significantly lower in Alberta (AB) (0.8%) and British Columbia (BC) (0.5%) than ON (1.8%; referent). Annual *B. burgdorferi* seroprevalence significantly increased between 2008 and 2015 in central, eastern and Atlantic Canada, with the greatest magnitude of change observed for MB (146.2%), ON (216.7%), QC (233.3%), and NS (441.7%); (all Cuzick test of trend: all *Z* > 8.60, all *P* < 0.001; Table 2). The complex province-specific temporal changes in *B. burgdorferi* seroprevalence were identified in the multivariable model (Table 3); year, year^2^, province and all two-way interactions were retained in the final model. Predictive probability plots of this model (Fig. 2) highlighted the significant increases in seroprevalence in ON, MB and QC. While seroprevalence appeared to be levelling by the end of the study period (2015) in ON (1.9%) and QC (3.0%), this was not observed in MB where seroprevalence was relatively high (3.2%) and continued to rise over the study period (Fig. 2).

**Table 3:** Multivariable logistic regression models for canine seroprevalence in Ontario, Manitoba and Quebec, Canada (2008–2015) for *Borrelia burgdorferi*, *Ehrlichia* spp. and *Anaplasma* spp., and the antigen of *Dirofilaria immitis*

Predictor	*Anaplasma* spp.		*B. burgdorferi*		*Ehrlichia* spp.		*D. immitis*	
	Coefficient (95% CI)	*P-*value	Coefficient (95% CI)	*P-*value	Coefficient (95% CI)	*P*-value	Coefficient (95% CI)	*P-*value
Year	0.35 (0.19–0.52)	<0.001	0.37 (0.30–0.45)	<0.001	0.26 (0.09–0.42)	0.003	-0.95 (-1.14– -0.75)	<0.001
Year^2^	-0.03 (-0.05– -0.02)	<0.001	-0.03 (-0.03– -0.02)	<0.001	-0.01 (-0.03–0.003)	0.1	0.09 (0.07–0.11)	<0.001
Province		<0.001		<0.001		<0.001		<0.001
Ontario	Referent		Referent		Referent		Referent	
Manitoba	0.82 (0.12–1.52)	0.02	0.93 (0.55–1.31)	<0.001	-2.29 (-3.80– -0.79)	0.003	-1.90 (-2.91– -0.88)	<0.001
Quebec	-1.57 (-2.72– -0.42)	0.007	-0.30 (-0.75–0.16)	0.2	-1.79 (-2.87– -0.70)	0.001	-3.41 (-4.69– -2.13)	<0.001
Province*Year		0.01		<0.001		0.03		<0.001
Ontario*Year	Referent		Referent		Referent		Referent	
Manitoba*Year	0.08 (-0.19–0.36)	0.6	-0.29 (-0.44– -0.13)	<0.001	0.75 (0.20–1.31)	0.008	1.07 (0.65–1.49)	<0.001
Quebec*Year	0.60 (0.17–1.03)	0.006	0.10 (-0.07–0.27)	0.3	0.85 (0.46–1.24)	<0.001	1.61 (1.12–2.10)	<0.001
Province*Year^2^		0.05		<0.001		<0.001		<0.001
Ontario*Year^2^	Referent		Referent		Referent		Referent	
Manitoba*Year^2^	-0.003 (-0.03–0.02)	0.8	0.03 (0.01–0.04)	<0.001	-0.06 (-0.11– -0.01)	0.02	-0.09 (-0.13– -0.05)	<0.001
Quebec*Year^2^	-0.05 (-0.09– -0.008)	0.02	-0.001 (-0.02–0.02)	0.9	-0.07 (-0.10– -0.04)	<0.001	-0.14 (-0.18– -0.09)	<0.001

**Fig. 3:**
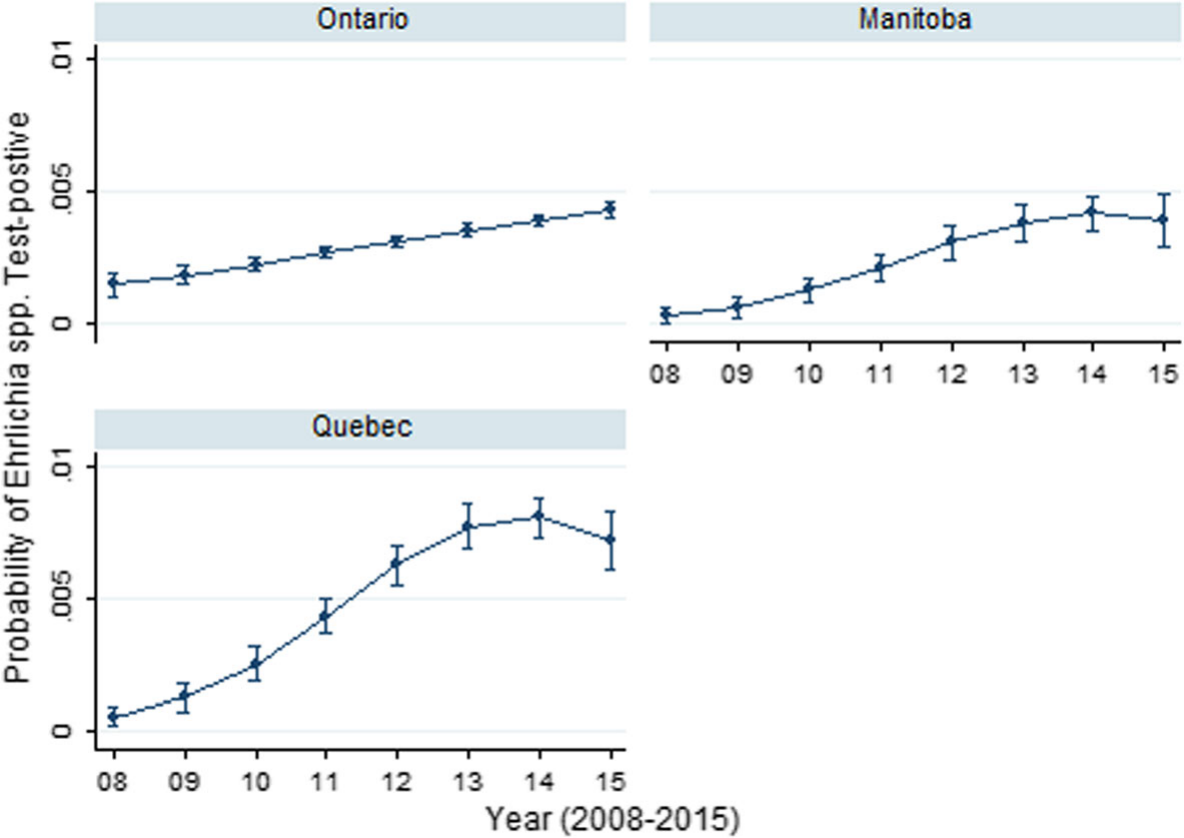
Predictive probability (with 95% CIs) of *Ehrlichia* spp. positive result on SNAP 4Dx tests performed on canine blood samples from dogs in Ontario, Manitoba and Quebec, Canada (2008–2015)

Seroprevalence for *Ehrlichia* spp. (Table 1) varied significantly among the provinces. The odds of *Ehrlichia* spp. seroprevalence was significantly greater than ON (0.4%; referent) in all provinces, with the exception of MB which was significantly lower (0.3%) (Table 1). This elevated seroprevalence was most evident in the western Canadian provinces of AB (4.9%) and BC (6.6%). Annual seroprevalence significantly increased between 2008 and 2015 in western (BC, AB), central (MB) and eastern Canada (ON, QC) (Table 2), particularly within Alberta, Ontario and Quebec (325%, 400% and 600% increase, respectively). The complex province-specific temporal changes in *Ehrlichia* spp. seroprevalence were identified in the multivariable model (Table 3); year, year^2^, province and all two-way interactions were retained in the final model. Predictive probability plots of this model (Fig. 3) highlighted the dramatic differences in seroprevalence across the study period in Ontario, Manitoba and Quebec. There were significant increases in *Ehrlichia* spp. seroprevalence in QC and MB (Fig. 3) with possible levelling of prevalence in 2014/2015. In contrast, a gradual linear increase was observed in ON (Fig. 3).

**Fig. 4:**
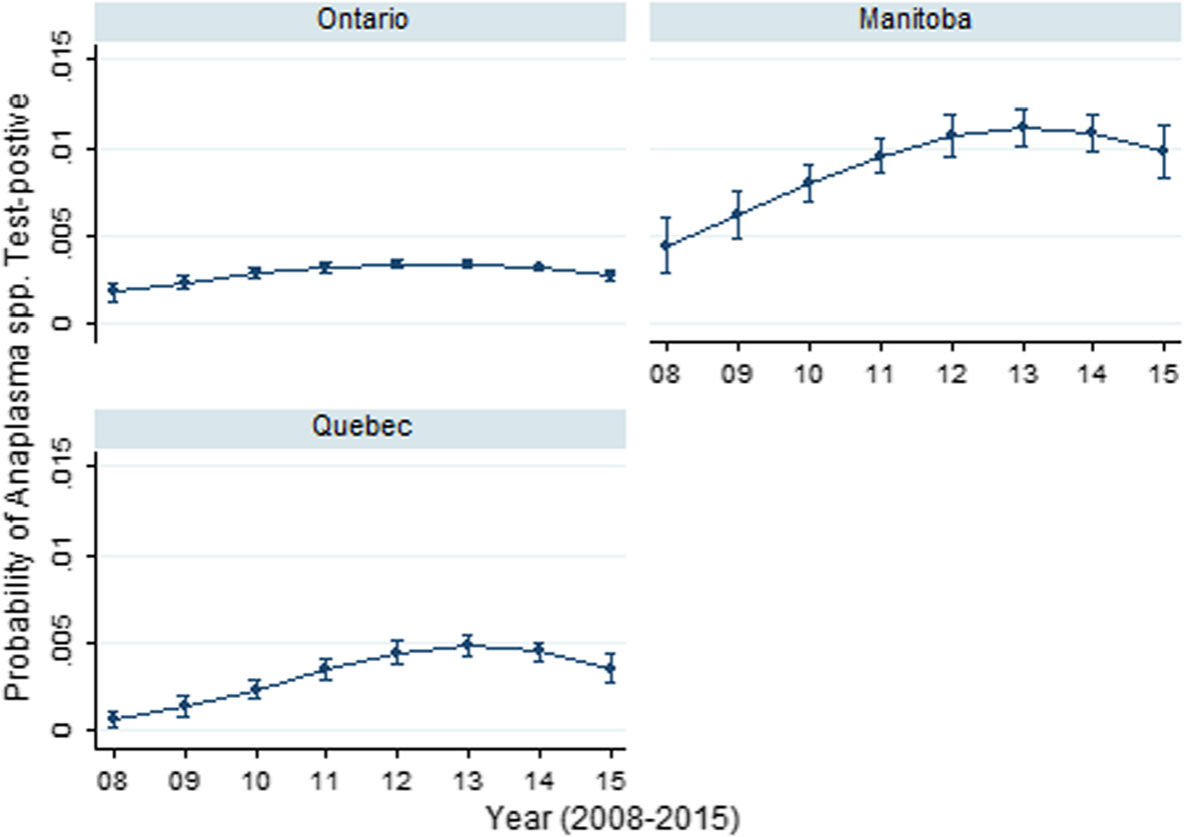
Predictive probability (with 95% CIs) of *Anaplasma* spp. positive result on SNAP 4Dx tests performed on canine blood samples from dogs in Ontario, Manitoba and Quebec, Canada (2008–2015)

Period seroprevalence for *Anaplasma* spp. (Table 1) varied significantly geographically. Odds of *Anaplasma* spp. seropositivity were higher in all provinces, except Saskatchewan (SK) and Newfoundland (NL), than in ON (0.3%; referent) (Table 1). Provinces with significant annual change, and the greatest magnitude of serological change (Table 2) were Manitoba and Quebec (233.3% and 185.7% positive increase, respectively). The province-specific temporal changes in *Anaplasma* spp. seroprevalence were identified in the multivariable model (Table 3); year, year^2^, province and all two-way interactions were retained in the final model. Predictive probability plots of this model (Fig. 4) highlighted the temporal differences in seroprevalence across the study period in Ontario, Manitoba and Quebec. There were initial increases with eventual levelling (2013–2015) in *Anaplasma* spp. seroprevalence in three provinces (ON, MB, QC). These findings were most pronounced in Manitoba.

**Fig. 5:**
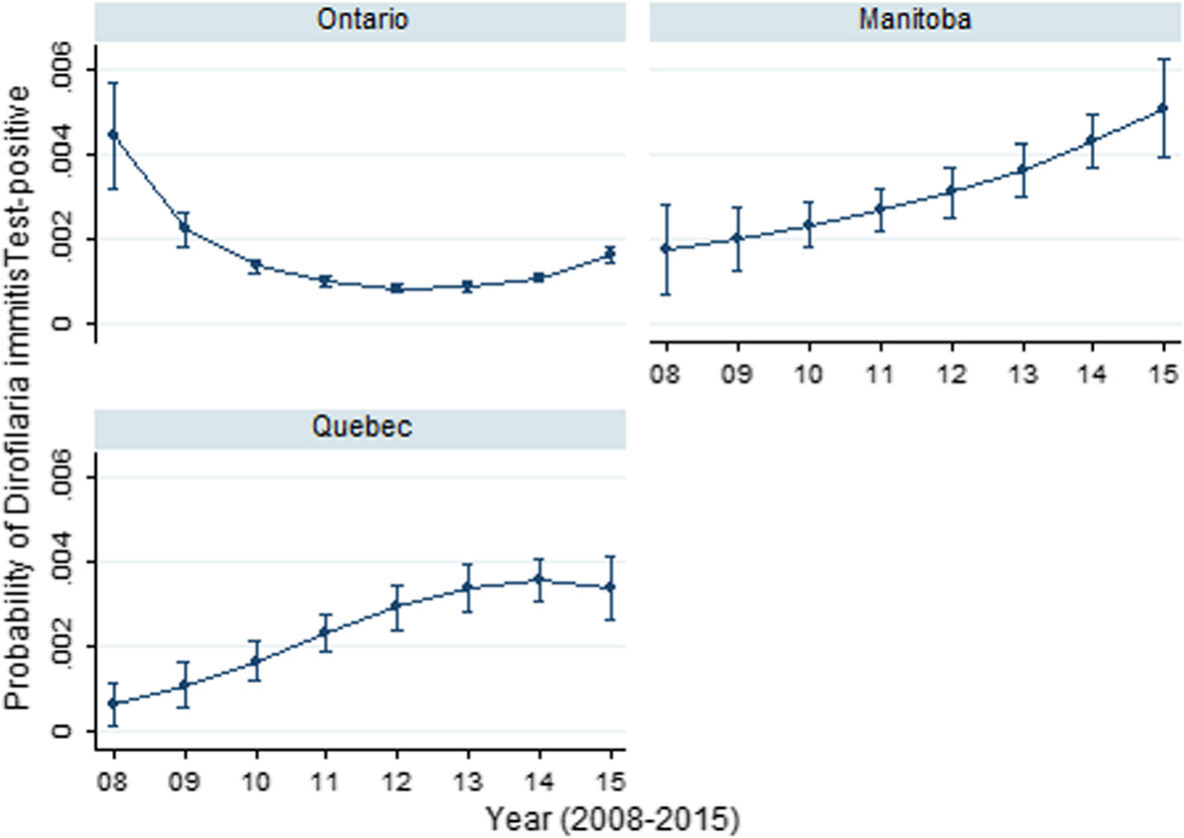
Predictive probability (with 95% CIs) of *Dirofilaria immitis* positive result on SNAP 4Dx tests performed on canine blood samples from dogs in Ontario, Manitoba and Quebec, Canada (2008–2015)

The odds of a dog being *D. immitis*-antigen positive were significantly greater in Quebec (0.3%) and Manitoba (0.4%) than Ontario (0.1%; referent) (Table 1). Over the study period, a significant positive annual trend in *D. immitis* antigen prevalence was noted in Manitoba (733.3% increase), while in Quebec, a significant annual decrease was observed (-25%). The changes in time in the predicted odds of a *D. immitis* antigen test varied among ON, MB, QC (Fig. 5); year, year^2^, province and all two-way interactions were retained in the final model. In Ontario, *D. immitis* prevalence showed a sharp decline before levelling off between 2013–2015, while Manitoba and Quebec revealed overall increasing trends in prevalence (Fig. 5).

## Discussion

The current study provides an important estimation of recent trends in canine vector-borne disease seroprevalence in Canada. *Borrelia burgdorferi* was the most common and widespread of the vector-borne pathogens that were evaluated. The seroprevalence noted here (2% over the 8-year study timeframe, 2.2% in 2015) was similar to a recent publication (2.5% nationally, 2013–2014) [5]. Not surprisingly, *B. burgdorferi* seroprevalences in our study were highest in Manitoba, Ontario, Quebec, New Brunswick and Nova Scotia. These are all provinces which contain recognized high-risk human Lyme disease regions [1, 3, 19–22]. The geographical variation and differences noted within Canada were expected, given disparities in national climate, which is a driving factor for *Ixodes scapularis* range and expansion [1–4]. The *B. burgdorferi* seroprevalence in Atlantic (NS), eastern (ON, QC) and central (MB) Canada displayed an overall increase over the 8-year study timeframe. The 2015 results from these higher seroprevalence regions are similar to adjacent regions in the United States [12, 13], and reported increased human Lyme disease incidence regions in Canada [19].

Canadian canine seroprevalence for *Anaplasma* spp. has been similarly evaluated and reported as below 0.5% (same test methodology) [5]. Until recently it was hypothesized that there was a very low risk of dog exposure for *Anaplasma* spp., specifically in Ontario [4, 23]. In contrast, another study performed in a region adjacent to Canada [13] found an increase in positive *Anaplasma* spp. serology in dogs from the Northeastern USA in 2010–2012 compared to 2001–2006 [12].

In dogs, anaplasmosis is due to exposure and infection with *Anaplasma phagocytophilum* or *A. platys* [11, 24]. Similar to *B. burgdorferi*, *A. phagocytophilum* is transmitted by *I. scapularis* and *I. pacificus* ticks [4, 11, 25]. However, *A. platys* is transmitted by *Rhipicephalus sanguineous*, a tick that is not believed to be established in Canada [4]. Due to their similar tick vector (*I. scapularis* and *I. pacificus*), *A. phagocytophilum* and *B. burgdorferi* may co-localize (i.e. tick infected with both pathogens) [26]. Tick co-localization of pathogens and the increasing number of *I. scapularis* ticks in these regions may provide an explanation for the annual prevalence increases in *Anaplasma* spp. seropositivity we noted in Manitoba, Nova Scotia, Quebec and Ontario. This finding may be further supported by the observed co-infections between *Anaplasma* spp. and *B. burgdorferi*. However, potential pathogen cross reaction in testing cannot be discounted and may, at least in part, be responsible for this increase [27].

In Canadian dogs, nation-wide *Ehrlichia* spp. seroprevalence has been reported as below 0.5% [5]. Other provincial studies have determined seroprevalence for *E. canis* as 3.2% (with 6/9 positive test results from Ontario) and 0% (*n* = 285) for *E. chaffeensis* or *E. ewingii* [14]. At present *E. canis* and *E. muris*-like agent (EMLA) appear to be the most likely clinically relevant *Ehrlichia* spp. in Canadian dogs. This is presumed due to their respective tick vector presence in the country, i.e. *Dermacentor variabilis* (*E. canis*) [28, 29] and *I. scapularis*, *I. cookei* (EMLA) [30, 31]. However, *D. variabilis* has only been shown to be experimentally infected with *E. canis*, so its role in natural infection and exposure in dogs is unclear [28]. Seroprevalence is increasing in the USA for *E. ewingii* [32], and it is considered an emerging vector-borne pathogen in dogs and humans. However, while both *R. sanguineus* and *A. americanum* have been reported in Canada [4] and can be infected with *E. ewingii*, neither are believed to be established ticks.

The change in prevalence for *Ehrlichia* spp. noted in Ontario and Quebec may be due to the presence of *I. scapularis-* and *I. cookei*-transmitted *E. muris*-like agent (EMLA), which has been documented in the adjacent mid-western USA region [30, 31, 33], *E. canis* or potentially due to *E. ewingii* vectored by emerging tick species. However, as neither *R. sanguineus* or *A. americanum* have been documented in Canada as established tick species [4] they would appear unlikely as vectors of *Ehrlichia* spp. to the extent we documented increasing seroprevalence in our study. As mentioned, cross-reaction can occur, and this may have resulted in some test misinterpretation and subsequent findings [27, 33].

Overall, the prevalence of heartworm was low and consistent with other studies [15]. However, a significant increase in prevalence was noted over the study time-frame in Manitoba. The range of competent mosquito vectors for this pathogen has been shown to have expanded northward [34], and other mosquito-borne pathogens are known to be present within this province [35]. This temporal rise in prevalence may also reflect a change in testing (e.g. increase in testing dogs with cardio-respiratory signs, recently imported dogs).

The tick vector of *B. burgdorferi* is known to carry other pathogens within Canada, and co-pathogen presence may complicate diagnosis, disease and therapy [8, 24, 36]. Co-infections were briefly described in an earlier Canadian study, with *B. burgdorferi* and *Anaplasma* spp. co-infection as 4.1%, and *B. burgdorferi* and *D. immitis* at 1.1% [15]. In this study, dogs with co-infection were more likely to be ill [15]. We estimated co-infections in 5% of all animals with at least one positive test, and this emphasizes the need for effective vector-borne disease prevention by Canadian veterinarians beyond reliance on vaccination for Lyme disease.

Limitations of prevalence studies such as the present work primarily relate to reporting bias, lack of travel history or confirmatory testing information on dogs [5, 12, 13]. Similarly, we cannot discount the possibility that differences in the proportion of tests being used for screening compared to clinical disease diagnoses may have resulted in the regional and temporal changes identified in seroprevalence. Further, although the tests used in this study have reported high sensitivities and specificities [27, 37–39], positive predictive values may be greatly affected by exposure and infection prevalence in specific geographies [40, 41]. The reported test specificity for *B. burgdorferi* using the SNAP 4Dx® Plus Test is 98.8% [27], and as such there is a potential for false positives. However, the geographical and temporal variations that were observed within this dataset, along with the multivariable analyses and predictive probabilities for Ontario, Manitoba and Quebec suggest that the specificity may be better than reported. As such, low prevalences are likely indicative of true positive results, rather than false positives.

In Canada, the distribution of several tick species is changing rapidly [1, 4, 42] and tick species emergence have been reported, e.g. *A. americanum*. Importation of livestock and companion animals, along with human travel with their animals have likely added to this distribution effect [43]. Emergence of ticks, range expansion and increased tick numbers have been documented, and this is most noteable with *I. scapularis* in central, eastern and Atlantic Canada [4, 21]. Similarly, albeit to a lesser extent, geographical range has increased for both *D. variabilis* and *D. andersoni* within central and western Canada [44]. The serological findings in our study echo this observed temporal and geographical change in tick vector range, and highlight the consequences of tick bite, attachment, subsequent exposure, infection and potential disease in dogs.

Significant differences among provinces in their pathogen period seroprevalences were observed, and significant trends over the course of the study within several provinces described. In provinces where we had sufficient power to conduct multivariable analyses, we noted that changes in prevalence over time were frequently not linear and the differences between provinces (i.e. Manitoba, Ontario, and Quebec) were not constant over time. These findings illustrate the importance of such modeling approaches, considering appropriate temporal and spatial units of measure, to allow for meaningful conclusions for these vector-borne pathogens. As testing increases throughout Canada, a similar approach will likely be useful to investigate these relationships in other Canadian provinces.

## Conclusions

This study builds on others performed in Canada and documents the prevalence of exposure and infection to common vector-borne pathogens in dogs, particularly the increase in prevalence of tick-borne pathogens. These geographical differences and temporal increases are most notable for *B. burgdorferi* in central, eastern and Atlantic Canada. However, significant positive increases were observed for *Ehrlichia* spp. (AB, ON, QC) and *Anaplasma* spp. (MB, ON, QC, NS). The substantial temporal changes in seroprevalence over the 8-year period observed within specific provinces highlights the rapid change in vector-borne pathogen presence. Ideally, the results of our findings will raise clinician awareness, increase canine and human tick prevention attention and prioritization, and reduce the risk of disease.

## Methods

### Study design

This repeated cross-sectional study utilized 753,468 test results for *D. immitis* antigen testing and *B. burgdorferi*, *Ehrlichia canis/ewingii/muris* serology, and 753,208 test results for *Anaplasma phagocytophilum/platys* serology submitted between 2008–2015. Data were obtained from an in-clinic ELISA test SNAP® 4Dx® (IDEXX Laboratories, Inc., Westbrook, Maine), and after 2012 the 4Dx® Plus Test (IDEXX Laboratories, Inc., Westbrook, Maine) tests that have been validated in dogs [27, 45]. Test results were generated by veterinary clinics and commercial veterinary diagnostic labs on dogs in Canada between 2008–2015. The majority of tests were analysed by the diagnostic laboratory, with a subset recorded manually in-clinic. Geography (clinic postal code) data were available. A small portion (*n* = 115,636, years 2013–2014) of the dataset overlaps with an earlier publication [5].

### Animals and households

Dogs were presumed to live with their owners and receive routine care through their veterinary clinic. Clinical data were not available, but it was presumed that most dogs were healthy and tested for the purpose of annual heartworm screening.

### Statistical analysis

Postal code of reporting veterinary practice was used to collate data by provinces. Univariable logistic regression was used to assess associations between a test result (outcome; performed for each of the four pathogens) and province. Odds ratios (OR) and the associated *P*-values were calculated for each pathogen. The non-parametric Cuzick test of trend was used for assessing associations between pathogen prevalence and year. Co-infections were visualized through a Venn diagram.

For provinces with sufficient positive and negative test results (Ontario, Manitoba, Quebec), multivariable logistic regression was used to assess the association between the odds of testing positive for these pathogens and the independent variables province and year. The linearity assumption for year was assessed by examining a lowess smoother plot of the log odds of the outcome against year, and by testing the statistical significance of the inclusion of a quadratic form for year (i.e. year^2^). If the relationship was not linear, year was modelled as a quadratic effect if appropriate, otherwise year was modeled as a categorical variable. A manual step-wise backward building procedure was used to create main-effects models. Prior to removal, likelihood ratio tests were used to assess the significance of each predictor. All two-way interactions between variables in the main-effects model (e.g. province, year, year^2^) were eligible for inclusion. Variables were retained in the final model if they were significant predictors for the given pathogen, part of a significant interaction term or acted as a confounding variable. A confounding variable was defined as a non-intervening variable whose removal resulted in a ≥ 20% change of another variable in the model [46]. Coefficients and 95% confidence intervals (CI) for the coefficients were reported for each pathogen. Annual predictive probabilities for a positive test result (along with 95% CIs) from final multivariable models were visualized through province-specific plots. Analyses were performed using commercial software (Stata version 13.1; StataCorp LP, College Station, TX). A significance level of 5% was used for all analysis.

## Additional files

Additional file 1: **Figure S1.** Percent positive results on SNAP 4Dx tests performed on canine blood samples from dogs in Ontario, Canada (2008–2015). (TIF 34 kb)

Additional file 2: **Figure S2.** Percent positive results on SNAP 4Dx tests performed on canine blood samples from dogs in Manitoba, Canada (2008–2015). (TIFF 35 kb)

Additional file 3: **Figure S3.** Percent positive results on SNAP 4Dx tests performed on canine blood samples from dogs in Quebec, Canada (2008–2015). (TIFF 34 kb)

## Abbreviations

BC: British Columbia
AB: Alberta
SK: Saskatchewan
MB: Manitoba
ON: Ontario
QC: Quebec
NB: New Brunswick
NS: Nova Scotia
PE: Prince Edward Island
NL: Newfoundland
OR: Odds ratio
95% CI: 95% confidence interval
