# Supplementary figures and images for: Prevalence of Borrelia burgdorferi, Anaplasma spp., Ehrlichia spp. and Dirofilaria immitis in Canadian dogs, 2008 to 2015: a repeat cross-sectional study
Source: Parasit Vectors. 2019 Jan 28;12:64. doi: 10.1186/s13071-019-3299-9 (PMC6350403; doi:10.1186/s13071-019-3299-9)

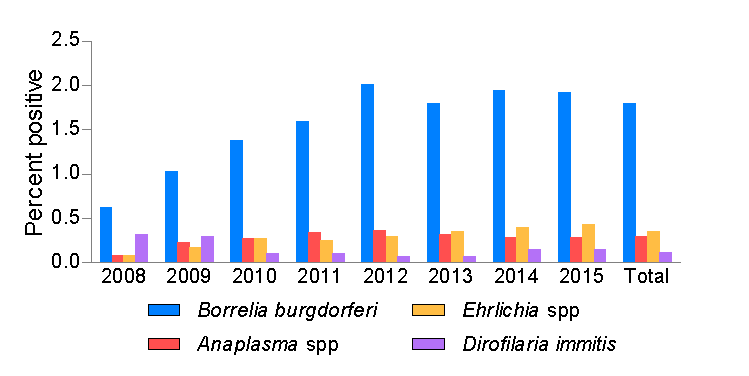

Supplement: Supplementary file 1 — Figure S1. Percent positive results on SNAP 4Dx tests performed on canine blood samples from dogs in Ontario, Canada (2008–2015). (TIF 34 kb) [file 13071_2019_3299_MOESM1_ESM.tif]

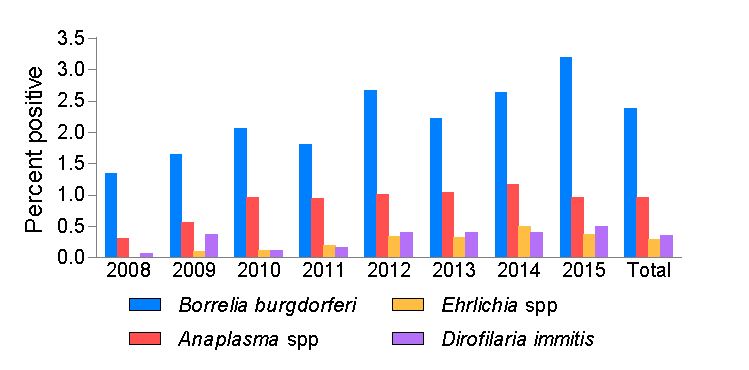

Supplement: Supplementary file 2 — Figure S2. Percent positive results on SNAP 4Dx tests performed on canine blood samples from dogs in Manitoba, Canada (2008–2015). (TIFF 35 kb) [file 13071_2019_3299_MOESM2_ESM.tiff]

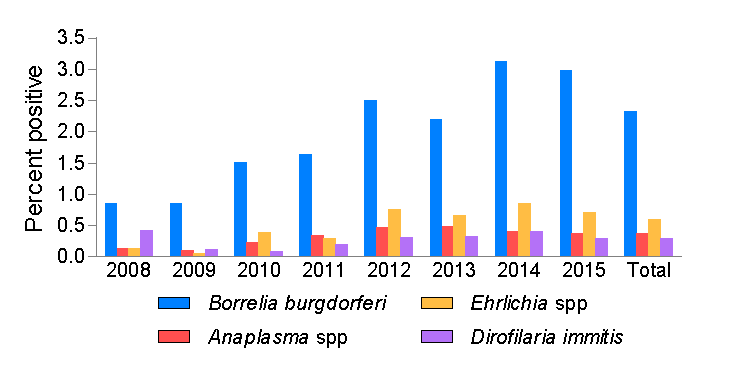

Supplement: Supplementary file 3 — Figure S3. Percent positive results on SNAP 4Dx tests performed on canine blood samples from dogs in Quebec, Canada (2008–2015). (TIFF 34 kb) [file 13071_2019_3299_MOESM3_ESM.tiff]
